# Proteomic and Phosphoproteomic Maps of Lung Squamous Cell Carcinoma From Chinese Patients

**DOI:** 10.3389/fonc.2020.00963

**Published:** 2020-06-16

**Authors:** Lulu Pan, Xijun Wang, Longhai Yang, Lei Zhao, Linhui Zhai, Junyu Xu, Yikun Yang, Yousheng Mao, Shujun Cheng, Ting Xiao, Minjia Tan

**Affiliations:** ^1^Chemical Proteomics Center, State Key Laboratory of Drug Research, Shanghai Institute of Materia Medica, Chinese Academy of Sciences, Shanghai, China; ^2^School of Pharmacy, University of Chinese Academy of Sciences, Beijing, China; ^3^State Key Laboratory of Molecular Oncology, Department of Etiology and Carcinogenesis, National Cancer Center/National Clinical Research Center for Cancer/Cancer Hospital, Chinese Academy of Medical Sciences and Peking Union Medical College, Beijing, China; ^4^Department of Thoracic Surgery, National Cancer Center/National Clinical Research Center for Cancer/Cancer Hospital, Chinese Academy of Medical Sciences and Peking Union Medical College, Beijing, China; ^5^Department of Cardiothoracic Surgery, Shenzhen University General Hospital/Shenzhen University Clinical Medical Academy, Shenzhen, China

**Keywords:** lung squamous cell carcinoma, proteomics, phosphoproteomics, clustering, lymph node metastasis, cell cycle

## Abstract

Lung squamous cell carcinoma (LUSC) is one of the leading causes of tumor-driven deaths in the world. To date, studies on the tumor heterogeneity of LUSC at genomic level have only revealed limited therapeutic benefits. Therefore, system-wide research of LUSC at proteomic level may further improve precision medicine strategies on individual demands. To this end, we performed proteomic and phosphoproteomic study for LUSC samples of 25 Chinese patients. From our results, two subgroups (Cluster I and II) based on proteomic data were identified, which were associated with distinct molecular characteristics and clinicopathologic features. Combined with phosphoproteomic data, our result showed that spliceosome pathway was enriched in Cluster I, while focal adhesion pathway, immune-related pathways and Ras signaling pathway were enriched in Cluster II. In addition, we found that lymph node metastasis (LNM) was associated with our proteomic subgroups and cell cycle pathway was enriched in patients with LNM. Further analysis showed that MCM2, a DNA replication licensing factor involved in cell cycle pathway, was highly expressed in patients with poor prognosis, which was further proved by immunohistochemistry (IHC) analysis. In summary, our study provided a resource of the proteomic and phosphoproteomic features of LUSC in Chinese patients.

## Introduction

Lung cancer is the most malignant tumor with the highest morbidity and mortality in the world (1, 2). Non-small cell lung cancer (NSCLC), as the most common histological types, accounts for more than 80% of all types of lung cancer, in which lung adenocarcinoma and lung squamous cell carcinoma (LUSC) are two major histopathological subtypes (3). In previous studies, LUSC was identified with multiple mutations in cancer driver genes such as *TP53* and *PTEN* (4), and four LUSC mRNA expression subtypes (primitive, classical, secretory, and basal) related with different biological processes (proliferation, xenobiotic metabolism, immune response, cell adhesion) were identified (5). However, there is still a lack of effective targeted therapies, except a few immunotherapies targeting at PD1 and PD-L1 (6–8). In contrast to genetic features, proteomic characteristics are more directive to reflect the pattern of LUSC as proteins are the “executioners of life” (9, 10).

Recently, a proteogenomic study on LUSC from Western patients has been conducted, which identified three proteomic subtypes associated with immune biology (inflammatory cluster), oxidation-reduction biology (redox cluster) and biology associated with Wnt/stromal signaling (mixed cluster). This study provided a resource and suggested therapeutic strategies based on metabolism and immune for LUSC in Western countries (11).

Global cancer statistics indicated that the occurrence of lung cancer has been decreasing in Western countries but increasing in developing countries (12, 13). In China, lung cancer ranks the first among all malignant tumors due to its high incidence and mortality rates (14). Preliminary researches indicated distinct genomic features of lung cancer for Chinese patients (15). For example, in NSCLC, *EGFR* mutation rate and *EGFR* mutational signatures associated with the inflammatory microenvironments were significantly higher in Chinese patients than those in Western patients. In terms of LUSC, Chinese patients had more frameshift indels in *CDKN2A* and more mutations in *NFE2L2*. Therefore, proteomic studies of LUSC on Western patients may not completely unveil the molecular features of LUSC at proteomic level from Chinese patients.

A previous proteomic study of 10 Chinese patients investigated the possible mechanism of bronchial epithelial carcinogenesis and identified several molecules for early detection, such as GSTP1, HSP1B, and CKB (16). However, systematic proteomic study of LUSC sample in large Chinese cohort is still limited. Besides, this previous work also did not investigate protein phosphorylation, an important protein post-translational modification essential for signaling conduction in cancer (17–23). Exploring molecular mechanisms on proteome and phosphoproteome of LUSC in a Chinese cohort will provide valuable information for the development of targeted therapy.

In this study, the proteomic and phosphoproteomic characteristics of LUSC samples in China were explored. Two subtypes based on proteomic and phosphoproteomic features were acquired. Combining clinicopathologic features, we unveiled that lymph node metastasis (LNM) was associated with clustering and related with patient prognosis possibly through cell cycle pathway.

## Materials and Methods

### Sample Collection

All biospecimens were obtained from the Cancer Institute/Hospital, Peking Union Medical College & Chinese Academy of Medical Sciences and Beijing Xuanwu Hospital, with the approval of the Research Ethics Committee at these two hospitals. By postoperative pathological analysis, these biospecimens were diagnosed as LUSC without other malignant tumors. All patients did not receive any radiotherapy, chemotherapy intervention or targeted therapy before surgery. The postoperative biospecimens were washed with physiological saline on ice to remove blood, and then directly frozen in liquid nitrogen for proteomic research. Judging tumor purity based on HE-stained slides, 25 LUSC tissues with tumor purity>50% were selected to build proteomic profiling and phosphoproteomic profiling. The clinical information was shown in Supplementary Table S1.

### Protein Extraction

Protein extraction was proceeded in refrigeration room to avoid protein digestion. The tissues were first washed by phosphate-buffered saline (PBS) and quickly dissected using surgical scissors. Then the total protein was extracted by 8 M Urea in 100 mM NH_4_HCO_3_ (pH 8.0) containing Protease Inhibitor (Roche) and Phosphatase Inhibitor (Roche) on ice for 30 min, followed by 3 min sonication under the condition of 3 s on and 5 s off with 30% power of JY92-IIN (NingBoXinZhi, China). Finally, the protein solution was collected after centrifugation, and the concentration was measured by BCA protein quantification kit (Beyotime Biotechnology, China).

### In-solution Digestion

Reaction of reduction was conducted at 56°C for 30 min, which was followed by alkylation reaction at 25°C for 30 min without light. Then the total protein was subjected to LysC (Mass Spec Grade, Hualishi Scientific) with a protease: protein ratio of 1:100 (w/w) for digestion at 37°C for 3 h. After being diluted by four times with 100 mM NH_4_HCO_3_ (pH 8.0), the protein was digested with trypsin (Mass Spec Grade, Hualishi Scientific) with a protease: protein ratio of 1:50 (w/w) at 37°C for 16 h. The digested peptides were then desalted by SepPak C18 cartridges (Waters, Milford, MA).

### TMT 6-Plex Labeling

Internal reference was adopted in the TMT labeling experiment. For the internal reference, 6 tumor samples including different clinical stages as well as differentiation were selected, the peptides were mixed in equal amount as the internal reference in each batch of TMT labeling experiments.

In TMT 6-plex labeling experiment, 200 μg peptides were labeled in every channel of each batch. The “internal reference” peptides were labeled with channel 126, and five tumor peptides were labeled with other channels 127–131, respectively. The isobaric labeling experiment was performed under the instruction of TMT kit. Briefly, TMT reagents were dissolved in ACN and added into peptides in 100 mM triethylammonium bicarbonate (TEAB). The labeling reaction was incubated for 1 h at room temperature, then the labeling reaction was quenched by 5% hydroxylamine for 15 min. After labeling efficiency test (the percentage of TMT modification at lysine residue and peptide N-termini >95%), the labeled peptides were combined at equal amounts and then desalted by SepPak C18 cartridges (Waters, Milford, MA). The 25 tumor samples were finally labeled into five batches in the TMT 6-plex experiment.

### HPLC Fractionation for Proteomic Analysis

TMT-labeled peptides (200 μg) in every batch were fractionated by reverse phase XBridge Prep C18 column (250×4.6 mm column containing 5 μm particles, Waters) using an Agilent 1100 HPLC System through a gradient from 3 to 90% buffer B (buffer A: 2% ACN, 98% H_2_O; buffer B: 98 % ACN, 2% H_2_O, the pH of both buffer A and buffer B was adjusted by NH_4_OH to pH = 10.0) with a flow rate of 1 ml/min for 90 min. Twenty fractions were obtained for every batch.

### TiO_2_ Enrichment for Phosphoproteomic Analysis

TMT-labeled peptides (1 mg) in each batch were subjected to enrichment by titanium dioxide (TiO_2_) as previously described (24). Briefly, TMT-labeled peptides were incubated with TiO_2_ beads (GL Science, Japan) in loading buffer (5% TFA, 70% ACN, 1 M lactic acid) at 25°C for 30 min. Peptides with nonspecific binding were washed away from beads with washing buffer (0.5% TFA, 70% ACN). Enriched phosphorylated peptides were eventually eluted with 4% ammonium hydroxide and fractionated by home-made C18 tip (3 μm particle size, Agela Technologies Inc.) into 6 fractions.

### LC-MS/MS Analysis

Proteomic and phosphoproteomic fractions were analyzed by Orbitrap Fusion following an EASY-nLC 1000 system (Thermo Fisher Scientific). A homemade reverse-phase C18 column (20 cm × 75 μm column containing 3 μm particle, Dikma Technologies Inc.) was used to separate peptides further through a gradient from 5 to 90% buffer B (buffer A: 0.1% FA in 2% ACN, buffer B: 0.1% FA in 90% ACN) in 70 min for proteomic analysis and in 110 min for phosphoproteomic analysis, respectively. Following nanoflow HPLC, Orbitrap precursor spectra were collected from m/z 450–1,500 (proteomic analysis) and m/z 350–1,500 (phosphoproteomic analysis) with a resolution of 60,000 at m/z 200, AGC of 5.0e5 and maximum injection time of 50 ms. In MS/MS acquisition, the top 15 precursors (proteomic analysis) and top 20 precursors (phosphoproteomic analysis) with intensity above 50,000 were selected to be fragmentized by Higher-energy Collision Dissociation with the normalized collision energy of 40%, then the fragment ions were detected in the Orbitrap with a resolution of 15,000 at m/z 200, the isolation window of 1 m/z, AGC of 5.0e5, dynamic exclusion of 50 s and maximum injection time of 80 ms.

### Database Searching

Raw files were processed by Proteome Discoverer (PD, version 2.2.0.388; Thermo Fisher Scientific) with the SEQUEST HT search engine against the UniProt human protein database (06/12/2018, 95,549 sequences) (25). TMT 6-plex was chosen as a method for quantification. Acetylation (+42.0105 Da) on protein N-termini and oxidation (+15.9949 Da) on Methionine (M) were designated as dynamic modifications. TMT 6-plex (+229.1629 Da) on Lysine (K) and peptide N-termini, and carbamidomethyl (+57.0215 Da) on Cysteine (C) were set as a static modification. For phosphoproteomic analysis, variable modifications also included phosphorylation (+79.9663 Da) on serine/threonine/tyrosine (S/T/Y). Trypsin/P was set as a specific enzyme with no more than two missed cleavages. The tolerances of MS and MS/MS were set at 10 ppm and 0.02 Da, respectively. The Percolator algorithm (26) in PD was adopted to control peptide spectrum matches at a false discovery rate (FDR) <1% and maximum delta Cn = 0.05. The cutoff of FDR at protein level was set as 1%. For identification of phosphosites, the localization probability threshold was set as 75% which was calculated by the ptmRS algorithm (27).

### Proteomic and Phosphoproteomic Data Analysis

#### Sample Quality Control and Data Normalization

Protein or phosphopeptide intensity was normalized by the median in each channel of five batches of TMT 6-plex experiments through total proteins to calibrate sample loading differences. The phosphosite and phosphoprotein intensity were derived from the sum of phosphopeptide and phosphosite intensity, respectively. For batch assessment, QC of internal reference was analyzed by Pearson correlation. To rule out abnormal samples, unimodal (Gaussian or normal) distribution was tested by a density plot of log_2_-transformed TMT ratios for the proteins. For data normalization, log_2_ TMT ratios for the proteins or phosphosites /phosphoprotein were normalized by z-score in each sample.

#### Proteomic and Phosphoproteomic Clustering

Robust proteomic clusters were derived by consensus clustering (28), using the proteins with (1) no missing values; and (2) the top 1,000 most varied proteins within twenty-four tumors. Robust phosphoproteomic clusters were derived by consensus clustering, using the phosphoproteins with (1) the number of missing values <20%; and (2) the top 1,000 most varied proteins within twenty-four tumors. For phosphoproteins, the missing values were imputated using a KNN algorithm. The data sets were clustered by k-means with *k* from 2 to 8 using the ConsensusClusterPlus R package. The consensus matrix, consensus CDF, delta area plot and silhouette plots were used to assess the appearance of different *k* values.

#### Pathway Enrichment Analysis

For proteomic data, gene set enrichment analysis (GSEA) (29) was conducted using gene set database “c2.cp.kegg.v6.2.symbols.gmt” from the MSigDB. For phosphoproteomic data, DAVID bioinformatics tool (30) was used to perform Kyoto Encyclopedia of Genes and Genomes (KEGG) pathway analysis (31).

### Immunohistochemistry (IHC) and Scoring

Paraffin-embedded LUSC tissue microarray (TMA) was purchased from Shanghai Outdo Biotechnology Company (Shanghai, China), which included 75 cases of LUSC patients with complete clinical pathology data and follow-up information. The TMA sections were baked at 65°C for 4 h and deparaffinized by xylene and ethanol, then incubated with 3% H_2_O_2_ for 10 minutes in the dark to remove endogenous peroxidase activity. After antigen retrieval by the citrate repair solution (pH = 6.0) in a microwave oven for 10 min, the sections were sealed with goat non-immune serum (MXB Biotechnology Company, Fujian, China) and incubated with a primary antibody for MCM2 or SAE1 at a 1:400 or 1:800 dilution (Abcam, UK) overnight at 4°C. Following incubation with the secondary antibody, DAB kit (MXB Biotechnology Company, Fujian, China) was applied for the chromogenic reaction. The sections were then counterstained with hematoxylin (Beijing solarbio science & technology Company, China). The staining results were analyzed and scored independently by two experienced pathologists. Based on the staining intensity and the positive percentage of tumor cells, samples were scored as four grades from 0 to 3 (0, negative; 1, weakly positive; 2, moderately positive; 3, strong positive). A score of 2/3 was defined as high protein expression and a score of 0/1 was defined as low protein expression.

## Results

### Global Profiling of LUSC Proteomics and Phosphoproteomics

**Figure 1:**
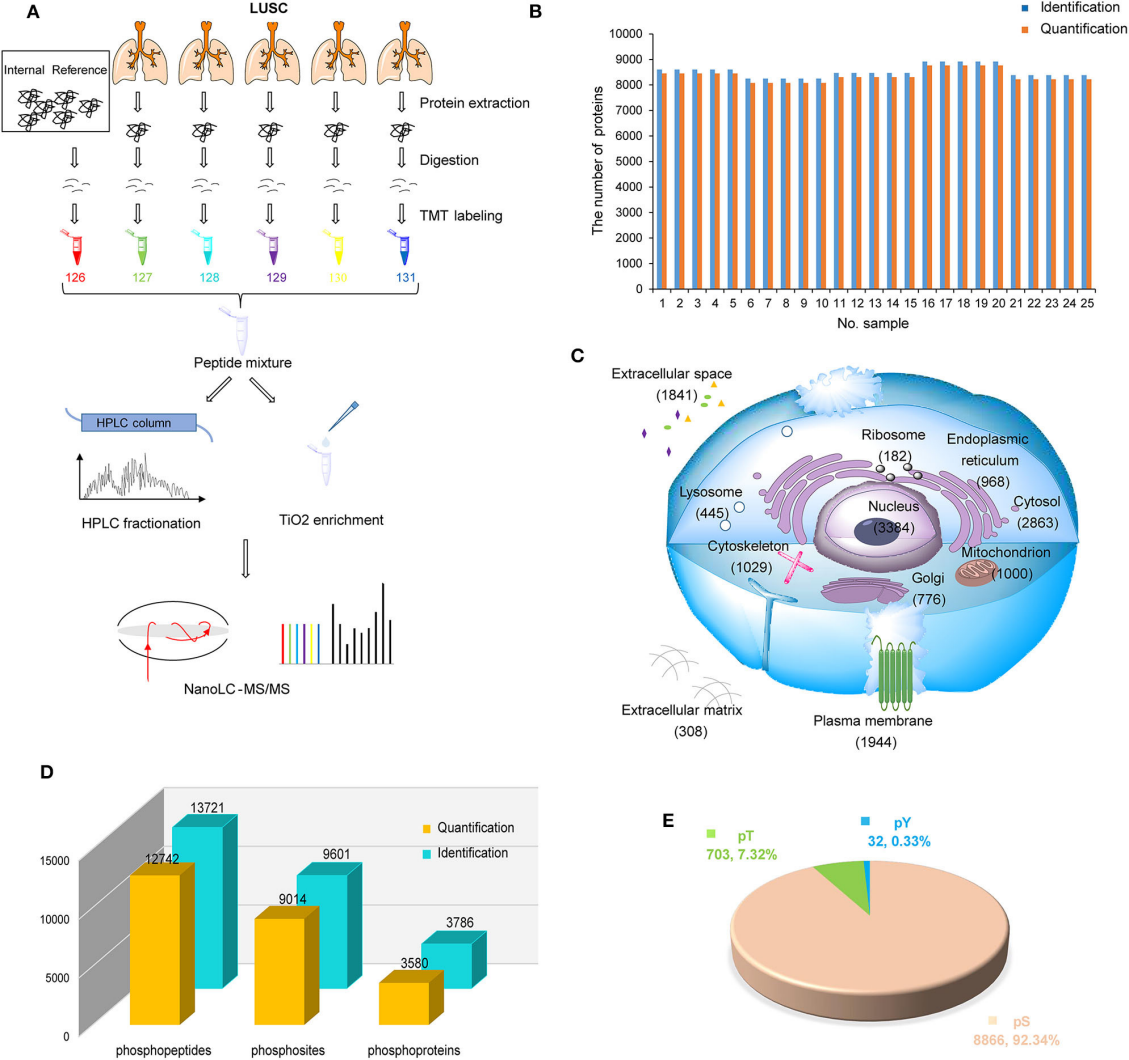
Proteomic and phosphoproteomic analysis of lung squamous cell carcinoma (LUSC). **(A)** Workflow of proteomic and phosphoproteomic profiling of LUSC. **(B)** The number of identified and quantified proteins in proteomic profiling. **(C)** Subcellular distribution of LUSC gene products annotated with Gene Ontology. **(D)** The number of identified and quantified phosphopeptides/sites/proteins in phosphoproteomic profiling. **(E)** Phosphosite distribution on S/T/Y phosphorylation residues.

To systematically investigate the characteristics of LUSC in China, LUSC tissues of 25 patients with tumor purity >50% were used to build proteomic and phosphoproteomic data (Figure 1A). After database searching by PD-SEQUEST HT (25, 32), 10,003 proteins were identified with high confidence (FDR < 1%), in which 9,907 were quantified. On average, 8,516 proteins per sample were identified, 8,360 proteins per sample were quantified (Figure 1B). A total of 6,523 proteins were quantified in all samples (Supplementary Table S2). To control the variation among batches, the proteomic data of five internal references from five batches were assessed using Pearson correlation. The results displayed high correlations among batches with an average Pearson correlation coefficient of 0.98 (Figure S1A, Supplementary Table S2). In order to rule out samples with abnormal distribution of protein abundance, the density plot of log_2_-transformed TMT ratios of proteins and dip statistic (33) were used to characterize these distributions, which showed that 25 samples were unimodal (Gaussian or normal) distribution (Figure S1B, Supplementary Table S3). After data normalization, each sample had a similar distribution with log_2_-transformed TMT ratios centered at zero without batch effect (Figure S1C, Supplementary Table S3). By subcellular distribution analysis through Gene ontology database from PANTHER14.1 (34), we found the most identified proteins were nuclear proteins, followed by cytoplasmic proteins and organelle proteins, which was consistent with Reference Proteomic dataset and could reflect the real distribution in tissue without subcellular preference. Most interestingly, a large number of proteins (1,841 proteins) were annotated as cellular components in extracellular space, suggesting that proteome in LUSC could be related to the tumor microenvironment (Figure 1C, Supplementary Table S4).

For phosphoproteomic analysis with database searching by PD-SEQUEST HT (27), 13,721 unique phosphopeptides (FDR < 1% and delta Cn < 0.05) and 9,601 unique phosphosites (localization probability > 0.75) on 3,786 proteins (FDR < 1%) were identified (Figure 1D, Supplementary Tables S5, S6). The distribution of phosphoserine (pS), phosphothreonine (pT) and phosphotyrosine (pY) sites was 92.34% (8,866), 7.32% (703), and 0.33% (32), respectively (Figure 1E, Supplementary Table S6). Similarly, correlation of phosphosites in five internal references were assessed to control variation among batches, which displayed high correlation with average Pearson correlation coefficient 0.789 (Figure S1D, Supplementary Table S7). After data normalization, each sample had a similar phosphosite distribution with log_2_-transformed TMT ratios centered at zero without batch effect (Figure S1E, Supplementary Table S7).

### Clustering Based on Protein Abundance

**Figure 2:**
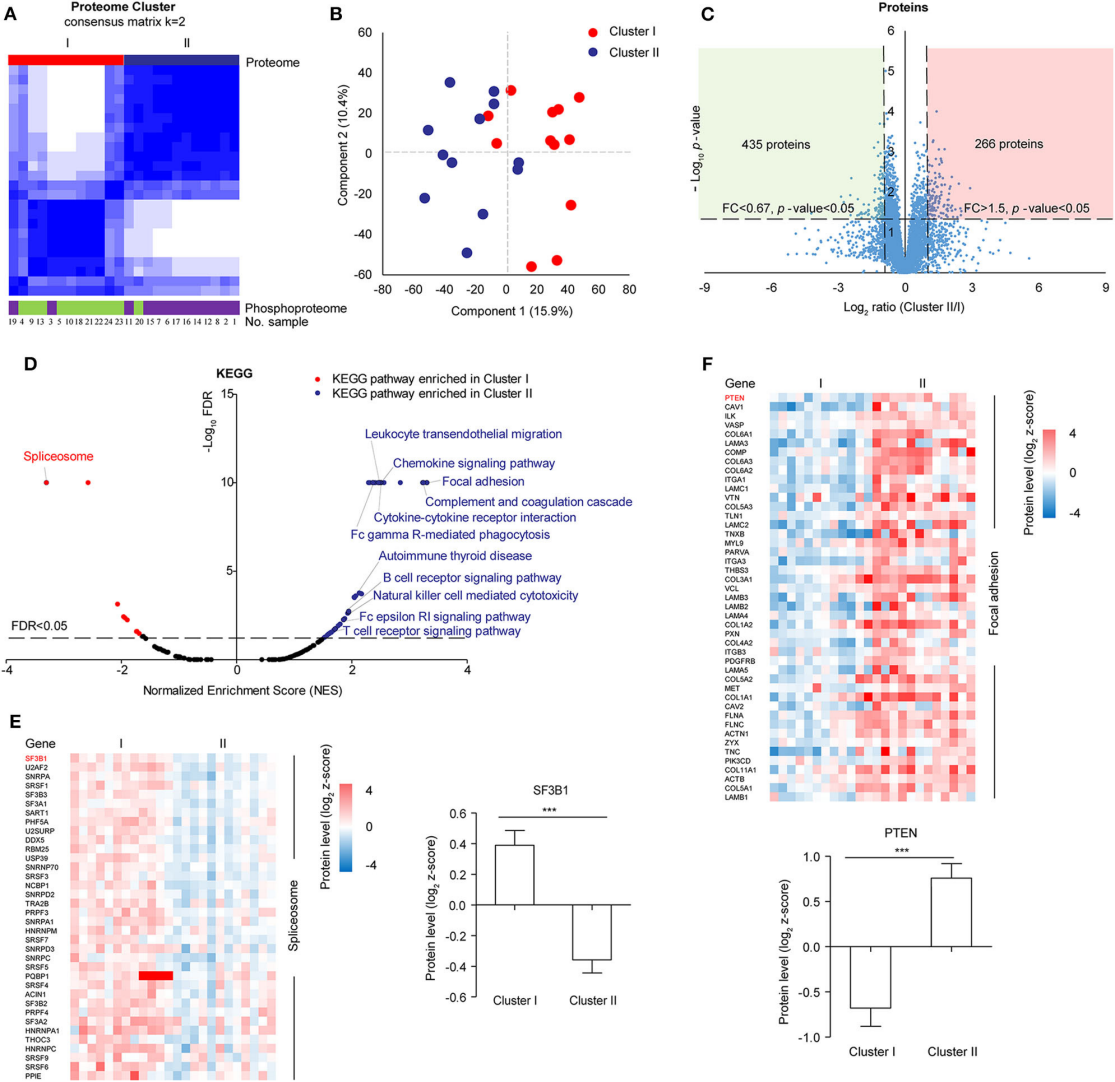
Proteomic clustering of LUSC. **(A)** Consensus-clustering analysis of proteomic profiling using the top 1,000 most varied proteins with no missing values within twenty-four tumors. Consensus-clustering analysis of phosphomic profiling using the top 1,000 most varied phosphoproteins with missing values <20% within twenty-four tumors. **(B)** Principal Component Analysis (PCA) of two proteomic clusters. Red represented Cluster I, and blue represented Cluster II. **(C)** Scatter plot depicting the fold change of protein abundance comparing cluster II with cluster I. Log_2_ fold changes were shown on the x-axis and –log_10_ *p*-values were shown on the y-axis. The vertical dashed lines indicated fold change > 1.5 and the horizontal dashed line indicated *p*-value < 0.05 (*t*-test). **(D)** GSEA analysis of proteomic data between cluster I and cluster II. The scatter plot showed the enriched KEGG pathways from the Molecular Signatures Database (MSigDB). Normalized enrichment score (NES) was shown on the x-axis and -log_10_ FDR was shown on the y-axis. The horizontal dashed line indicated FDR < 0.05. The labeled pathways were the most significant pathways or pathways consistent with previous data reported in samples from Western countries. **(E)** Differential protein expression of cluster I and cluster II in spliceosome. **(F)** Differential protein expression of cluster I and cluster II in focal adhesion.

In the follow-up analysis, a sample with incomplete clinical information was removed, the remaining 24 samples were clustered by consensus clustering using k-means manner to explore the proteomic difference among LUSC tissues. Visually, the consensus matrix for *k* = 2 appeared to have the cleanest separation between clusters (Figure 2A, Figure S2A). The consensus CDF and delta area plot showed that there was no significant increase in the area under the consensus CDF as *k* increased from two (Figure S2B). Furthermore, the average silhouette distance for *k* = 2 (0.14) was larger than *k* = 3 (0.09). In addition, phosphoproteomic clustering analysis was also performed, in which the top 1,000 most varied phosphoproteins with less than 20% missing values within twenty-four tumors were used because the number of identified phosphoproteins were much less than the number of identified of proteins. The phosphoproteomic clustering was almost consistent with proteomic clustering (Figure 2A, Figures S3A,B). Therefore, 24 samples can be clustered to Cluster I (*n* = 12) and Cluster II (*n* = 12). The clustering results were then verified by principal component analysis, in which component 1 and component 2 accounted for 26.3% of the total data variation and the two components can distinguish Cluster I and Cluster II (Figure 2B).

For 6,523 proteins which were quantified in all the 24 samples (Supplementary Table S8), 435 proteins were significantly upregulated in Cluster I and 266 proteins were significantly upregulated in Cluster II (fold change> 1.5 and *t*-test *p*-value < 0.05) (Figure 2C). Gene set enrichment analysis (GSEA) showed that spliceosome pathway was enriched in Cluster I, focal adhesion and immune-related pathways (e.g. Complement and coagulation cascades) were enriched in Cluster II (Figure 2D, Supplementary Table S9). For spliceosome, various studies have highlighted the significance of altered RNA splicing in cancer (35). The leading edge proteins in the spliceosome pathway were major spliceosome components and expressed higher in Cluster I, including SF3B1 (splicing factor 3b subunit 1) which was identified with recurrent mutations in various cancer (Figure 2E) (36, 37). In focal adhesion, some of the leading-edge proteins were constituents which participate in the structure linking membrane receptors and the actin cytoskeleton, while others contribute to signal transduction, including protein kinases and phosphatases (e.g., PTEN) (Figure 2F). Immune-related pathways (e.g., complement and coagulation cascades) were also enriched in Cluster II, which was similar to “inflammatory” subtype in previous data reported in samples from Western countries (11).

### Phosphoproteomic Analysis Based on Clustering

**Figure 3:**
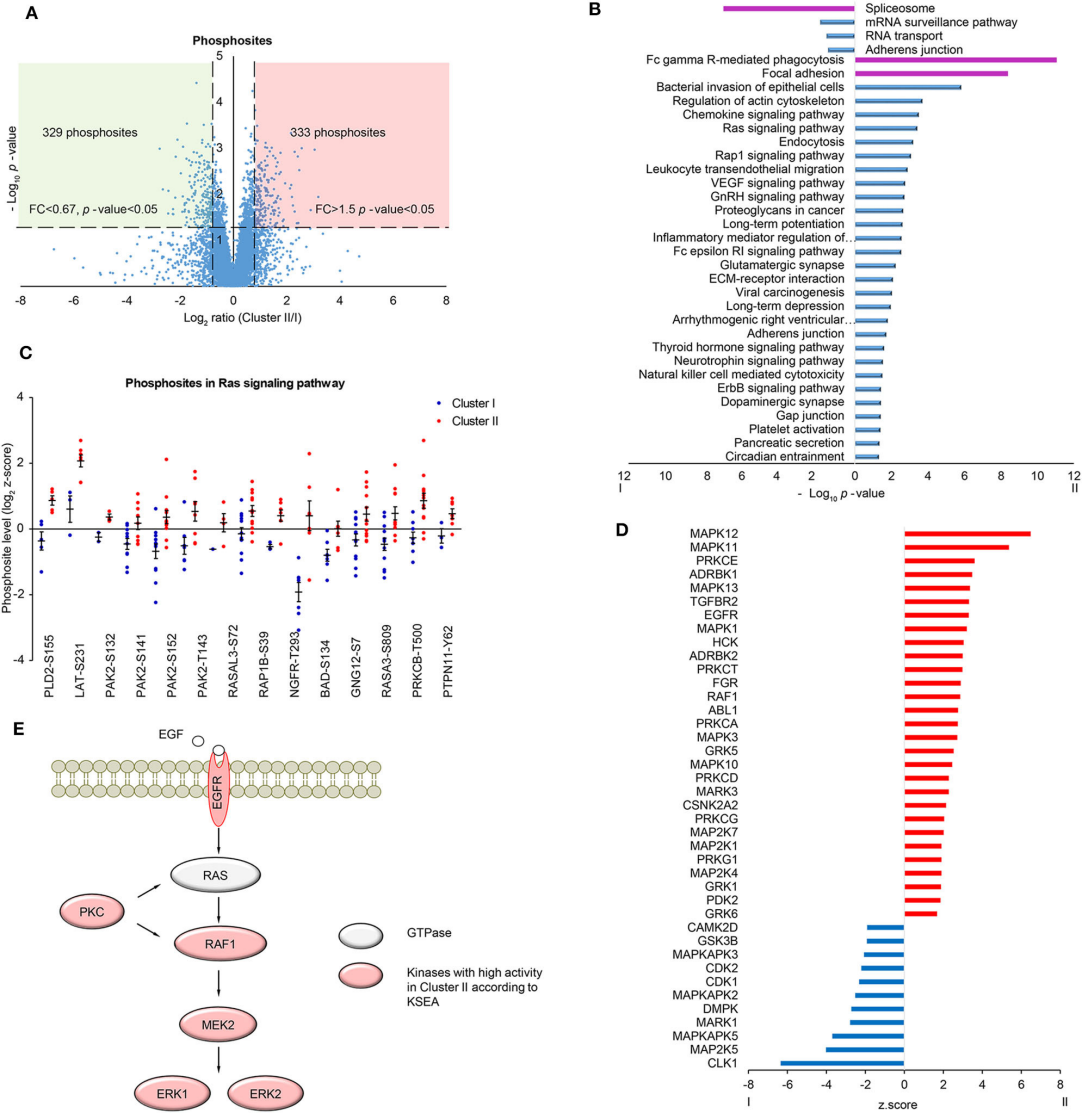
Phosphoproteomic analysis of LUSC comparing cluster I with cluster II. **(A)** Scatter plot depicting the fold change in phosphosites comparing cluster II with cluster I. Log_2_ fold changes were shown on the x-axis and -log_10_ *p*-values are shown on the y-axis. The vertical dashed lines indicated fold change > 1.5 and the horizontal dashed line indicated *p*-value < 0.05 (*t*-test). **(B)** DAVID analysis of phosphoproteomic data between cluster I and cluster II (*p*-value < 0.05). The bar chart showed the enriched KEGG pathways of the differential phosphoproteins between cluster I and II (fold change > 1.5, *p*-value < 0.05). The highlighted pathways were consistent with those enriched in protein level (purple). **(C)** The quantification of phosphosites in Ras signaling pathway. Data were presented as dot plot with mean ± SEM. **(D)** Kinase-substrate enrichment analysis (KSEA) based on PhosphoSitePlus and NetworKIN database. Color blue/red for visual annotation of kinases that reached statistical significance (*p*-value < 0.05). **(E)** A scheme showing the enriched kinases in the MAPK pathway.

In order to further investigate the differences in signaling pathways between the two clusters, phosphoproteomic data including 7,973 phosphosites (at least two values in each cluster, Supplementary Table S10) was analyzed. The scatter plot showed that 329 phosphosites on 224 proteins were significantly upregulated in Cluster I and 333 phosphosites on 264 proteins were significantly upregulated in Cluster II (fold change> 1.5 and *t*-test *p*-value < 0.05) (Figure 3A). Then proteins with up/down-regulated phosphosites were used for KEGG pathway analysis via DAVID. Consistent with proteomic analysis, spliceosome was enriched in Cluster I, immune-related pathways and focal adhesion were enriched in Cluster II (Figure 3B, Supplementary Table S11). Besides, several proteins in Ras signaling pathway showed highly phosphorylated sites in Cluster II, including BAD Ser-134 (Figure 3C). BAD Ser-134 was reported to be phosphorylated by RAF, which leads to increased proliferation of cancer cells (38). In the cascade of signaling pathway, kinase activity plays an important part by regulating the change of phosphosite level. Here, we found the potential change of activity in several kinases by Kinase-Substrate Enrichment Analysis (KSEA) (Figure 3D, Supplementary Table S12) (39, 40). Among these, MAPKAPK2/3/5 and CDK1/2 showed higher kinase activity in Cluster I, whereas EGFR, PRKCA/G, MAPK1/3/10/11/12/13, and MAPK2K1/4/7 showed higher kinase activity in Cluster II. Different kinases in MAPK pathway may be involved in different biological processes, for example, MAPKAPK family corresponds to TNF stimulation and most of MAPK family corresponds to GF stimulation (41, 42). Further analysis discovered that several kinases were important components in MAPK signaling pathway and displayed in the scheme (Figure 3E).

### Analysis of Lymph Node Metastasis (LNM)

**Figure 4:**
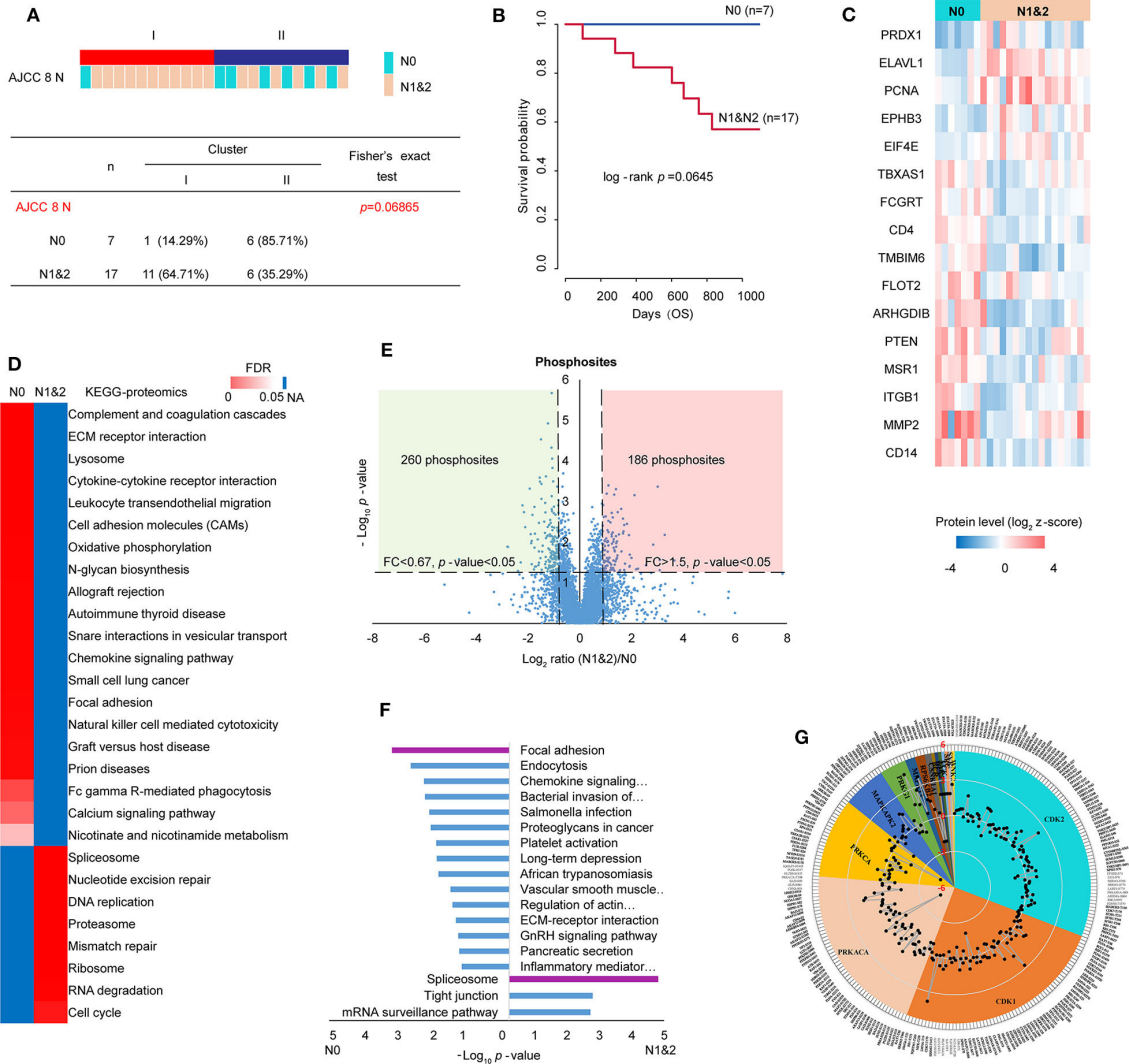
Analysis of lymph node metastasis (LNM). **(A)** Relationship of cluster with LNM. Fisher's exact test was adopted for the analysis. **(B)** The prognosis of patients with or without LNM within 3-year follow-up. **(C)** 16 differential metastasis-related proteins based on total 355 proteins which were associated with lung cancer metastasis from HCMDB database (fold change> 1.5, *p*-value < 0.05). **(D)** GSEA analysis of proteomic data with and without LNM (tumor samples, *n* = 24): N0 (without LNM, *n* = 7) and N1&2 (with LNM, *n* = 17). The heatmap showed the enriched KEGG pathways from MSigDB. The color in heatmap was according to FDR, and the darkest blue represents N/A. **(E)** Scatter plot depicting the fold change in phosphosites comparing N1&2 with N0. Log_2_ fold changes were shown on the x-axis and –log_10_ *p*-values are shown on the y-axis. The vertical dashed lines indicated fold change > 1.5 and the horizontal dashed line indicated *p*-value < 0.05 (*t*-test). **(F)** DAVID analysis of phosphoproteomic data between N1&2 and N0. The bar chart showed the enriched KEGG pathways of the regulated phosphoproteins between N1&2 and N0 (fold change> 1.5, *p*-value < 0.05). The highlighted pathways were consistent with those enriched in the protein level. **(G)** Circular plot represented potential kinases and phosphosites retrieved from the PhosphoSitePlus database. The phosphosites were differentially expressed between N1&2 and N0 with a cutoff of 1.5-fold change. Different colors correspond to various kinases that were predicted as upstream regulators of the phosphosites (*p*-value < 0.05). The outer circle shows the sites. The radar map shows the fold changes of the sites.

Clinicopathologic characteristics are important factors which could be related to molecular features and patient prognosis. Therefore, Clinical information of the proteomic set samples was linked with clustering (Supplementary Table S13). Compared with Cluster II, Cluster I showed a little poor prognosis with log-rank *p*-value 0.2 within 3-year follow-up (Figure S4A). Although most clinicopathological characteristics showed no significant relationship with clustering statically, it was prominent that lymph node metastasis (LNM) showed some correlation with clustering (Fisher's exact test *p*-value 0.069) (Figure 4A). In detail, 6 of 7 patients without LNM (N0) were in cluster II and 11 of 17 patients with LNM (N1&2) were in cluster I. Patients with LNM showed a little poor prognosis (log-rank *p*-value 0.0645) within 3-years follow-up (Figure 4B, Figure S5A). Tumors often engage the lymphatic system to invade and metastasize (43). LNM is an indication of poor prognosis (44). Together, all the evidence indicated that the study on LNM in LUSC was valuable. To identify metastatic proteins, we took advantage of the Human Cancer Metastasis Database (45). There are 355 proteins associated with lung cancer metastasis in HCMDB, among which, 16 showed differential expression between N0 and N1&2 (fold change> 1.5 and *t*-test *p*-value < 0.05) (Figure 4C). In detail, 5 proteins [e.g., PRDX1 (peroxiredoxin 1) and ELAVL1 (ELAV like RNA binding protein 1)] were highly expressed in N1&2, whereas other 11 proteins (e.g., PTEN) were highly expressed in N0, indicating that these proteins may also play a role on LNM in LUSC.

In order to explore the pathways associated with LNM, the relative expression of 6,523 proteins (no missing value in 24 samples) were used to conduct KEGG analysis by GSEA. The result showed that immune-related pathways and focal adhesion were enriched in N0, cell cycle-related pathways and spliceosome were enriched in N1&2 (Figure 4D, Supplementary Table S14). To further investigate the differences in signaling pathways between N0 and N1&2, phosphoproteomic data including 7,209 phosphosites (at least two values in each cluster, Supplementary Table S15) was analyzed. The scatter plot showed that 260 phosphosites on 221 proteins were significantly upregulated in N0 and 186 phosphosites on 140 proteins were significantly upregulated in N1&2 (fold change > 1.5 and *t*-test *p*-value < 0.05) (Figure 4E). The proteins with differential phosphosites were used to enrich KEGG pathways via DAVID. Focal adhesion, as well as immune-related pathways, were enriched in N0, spliceosome was enriched in N1&2 (Figure 4F, Supplementary Table S16). We next explored upstream kinases enriched by the phosphosites in KSEA using the PhosphoSitePlus database (46) (Supplementary Table S17). This analysis revealed that 4 kinases (PRKACA, PRKCA, CSNK1A1 and BCR) showed higher kinase activity in LUSC without LNM, whereas the other 12 kinases showed higher kinase activity in LUSC with LNM, including cyclin-dependent kinase 1 (CDK1) and cyclin-dependent kinase 2 (CDK2) which are key regulatory enzymes in cell cycle, indicating that cell cycle may be potentially activated on the condition of LNM (Figure 4G).

### Cell Cycle and DNA Replication in LUSC Based on LNM

**Figure 5:**
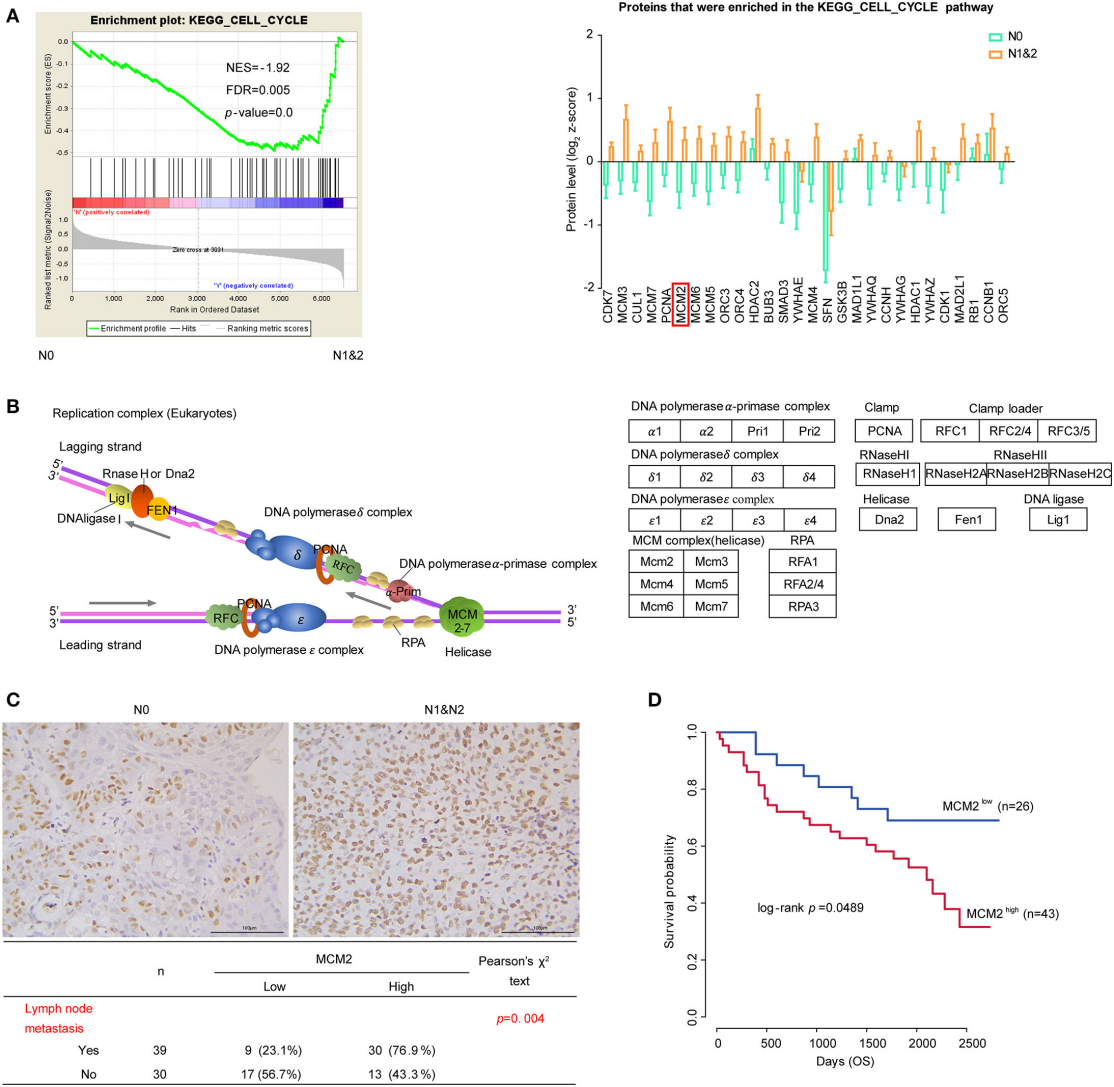
Cell cycle and DNA replication in LUSC based on LNM. **(A)** Cell cycle pathway and proteins that were enriched in the pathway. The protein quantifications are presented as mean ± SEM. **(B)** The diagram of DNA replication in cell cycle. Different protein complexes are represented in different colors. **(C)** The relationship MCM2 protein expression and LNM in tissue microarray (TMA) assay. **(D)** MCM2 expression and survival analysis in tissue microarray (TMA) assay. Chi-square test was adopted for the correlation analysis, and Kaplan–Meier plot (Log-rank test) was adopted to describe overall survival (OS) analysis.

Cancer is characterized by uncontrolled tumor cell proliferation resulting from aberrant activity of various cell cycle proteins. Therefore, cell cycle regulators are considered attractive targets in cancer therapy (47). In our data, cell cycle pathway was significantly enriched in the N1&2 group. The expressions of the leading edge proteins which contributed most to the enrichment score (ES) were upregulated with LNM (Figure 5A). Among these proteins, most were associated with DNA replication, as shown in the diagram (Figure 5B). DNA replication licensing factors- Minichromosome Maintenance (MCM) are essential for initiating and limiting DNA replication in cell cycle and implicates prognostic significance in lung cancer (48, 49). All members in the MCM protein family were highly expressed in the N1&2 group as well as in Cluster I (Figure 5A, Figure S5C). MCM2 is an independent predictor of survival in patients with non-small-cell lung cancer (50). To explore the influence of MCM2 on LUSC, we conducted IHC analysis based on TMA including 75 cases of LUSC patients, we found that MCM2 had higher expression in LUSC with LNM (Figure 5C, Supplementary Table S18). These findings suggested that high MCM2 expression in LUSC could be related to high biological malignant aggressiveness. Further survival analysis indicated that high expression of MCM2 was associated with poor prognosis (log-rank *p*-value = 0.0489) (Figure 5D). These results suggested that MCM2 might be a potential therapeutic target for LUSC, especially for LUSC with LNM.

## Discussion

LUSC accounts for a significant percentage of NSCLC (about 40%), but has limited biomarkers for diagnosis and therapy, except for a few immunotherapies targeting at PD1 and PD-L1 (6–8). In addition, systematic study on the molecular mechanism of Chinese LUSC patients is limited. Further study on the molecular mechanism of LUSC is needed for targeted therapy, especially in the Chinese cohort. To explore the protein expression pattern and the activation of signaling pathways in cancer, we investigated the proteomic and phosphoproteomic characteristics of LUSC from Chinese patients based on mass spectrometry analysis. In order to distinguish molecular characteristics, the samples were clustered into two parts on the basis of proteomic profiling. Compared with previous data reported in samples from Western countries (5, 11), our data showed that immune-related pathways were enriched in Cluster II, which was similar to the “inflammatory” subtype at protein level and “secretory” subtype at mRNA level. Even though the “redox” was not a major pattern in Cluster I, PSAT1 (phosphoserine aminotransferase), a potential target in the “redox” subtype, was highly expressed in this cluster (Figure S4B) (11, 51). These results suggested that there were some similar patterns across the races. Beyond the previous study, our data indicated that spliceosome pathway was enriched in cluster I, focal adhesion pathway was enriched in cluster II in both proteomic and phosphoproteomic data.

In the pathway analysis of proteomic data, we found one-ubiquitin mediated proteolysis was enriched in Cluster I with FDR 0.18 (Figure S4C), and PPI network of the leading edge proteins analyzed by STRING revealed that most proteins were closely associated with the surrounding proteins in the network. Among these proteins, SAE1 was reported to be highly expressed in a variety of cancers and promotes tumor progression as well as poor prognosis (52–55). To explore the influence of SAE1 on LUSC, we conducted IHC analysis based on TMA including 75 cases of LUSC patients, which showed that high expression of SAE1 was associated with poor prognosis (Figure S4D, Supplementary Table S16). In the pathway analysis of phosphoproteomic data, Ras signaling pathway was highly activated in Cluster II and the activity of several kinases was shown to be elevated in Cluster II compared with Cluster I, indicating Ras signaling pathway may be different in these two clusters.

In order to comprehensively analyze molecular features and clinicopathologic characteristics, we first linked clinicopathologic characteristics with clustering, which showed some correlation between LNM and clustering. Patients with LNM showed a little poor prognosis. Considering the lymphatic system was often engaged by tumor invasion and metastasis (43), we supposed that LNM should have a significant influence on prognosis with a larger sample size, which was confirmed by analyzing public data from the previous study (Figure S5B) (11). Both proteomic and phosphoproteomic data were used for pathway analysis and showed enrichment of several major pathways in N0 (or N1&2), similar to those found in Cluster II (or Cluster I). Besides, key regulatory enzymes in cell cycle were also enriched in LUSC with LNM by KSEA.

DNA replication is a key in cell cycle and MCM2 was discovered with high expression in patients with LNM by proteomics analysis and in patients with poor prognosis by IHC analysis. MCM2 is one of the members of the MCM protein family. It forms MCM complex with its family members MCM3-7. The MCM complex is a replication helicase, which is essential for the DNA replication initiation and extension of cell cycle in eukaryotic cells (56). The expression level of MCM proteins (MCMs) in normal cells changes with the progress of the cell cycle. In the G1 phase of the cell cycle, CDK activates the transcription factor E2F by phosphorylating RB, E2F can combine with the promoter region of MCMs to promote its transcription (57, 58). In senescent cells, p53 can synthesize microRNAs to degrade MCMs mRNA (59). In summary, MCM proteins could be dysregulated by different signaling pathways in cancer. In our proteomic data, cyclin-dependent kinases (CDK1/2) showed higher kinase activity in LUSC with LNM which may regulate MCM2 expression. However, The Cancer Genome Atlas (TCGA) dataset showed the RNA level of MCM2 was not correlated with prognosis in LUSC (Figure S5D) (6), which suggested there may be different features in genomics and proteomics. Further, MCM2 was shown to be not correlated with prognosis in LUSC from Western patients (Figure S5E) (11), which indicated there may exist different mechanisms among races. In our IHC analysis, MCM2 displayed higher expression in advanced patients (Figure S5F). Altogether, our study provided a proteomic and phosphoproteomic data resource about LUSC from Chinese patients, which could give several clues on potentially targeted proteins for precision medicine.

## Data Availability Statement

The datasets presented in this study can be found in online repositories. The names of the repository/repositories and accession number(s) can be found below: the integrated Proteome resources (iProx database) (http://www.iprox.org/index), accession number IPX0001833000.

## Ethics Statement

The studies involving human participants were reviewed and approved by the Ethics Committee of the Cancer Institute/Hospital, Peking Union Medical College & Chinese Academy of Medical Sciences and Beijing Xuanwu Hospital. The patients/participants provided their written informed consent to participate in this study. Written informed consent was obtained from the individual(s) for the publication of any potentially identifiable images or data included in this article.

## Author Contributions

LP, XW, and LY conducted most of the proteomic and biochemical experiments with the help of YY, YM, and SC under the supervision of TX and MT. LP, XW, and LY analyzed the data with the help of LZhao, LZhai, and JX under the supervision of TX and MT. LP and XW wrote most of the manuscript with the guidance and help of TX and MT revised it. All authors read and approved the final manuscript.

## Conflict of Interest

The authors declare that the research was conducted in the absence of any commercial or financial relationships that could be construed as a potential conflict of interest.
