# Supplementary material for: Proteomic and Phosphoproteomic Maps of Lung Squamous Cell Carcinoma From Chinese Patients
Source: Front Oncol. 2020 Jun 16;10:963. doi: 10.3389/fonc.2020.00963 (PMC7308564; doi:10.3389/fonc.2020.00963)
Supplement: Supplementary file 2 [file Image_1.pdf]

Supplementary Figure 1

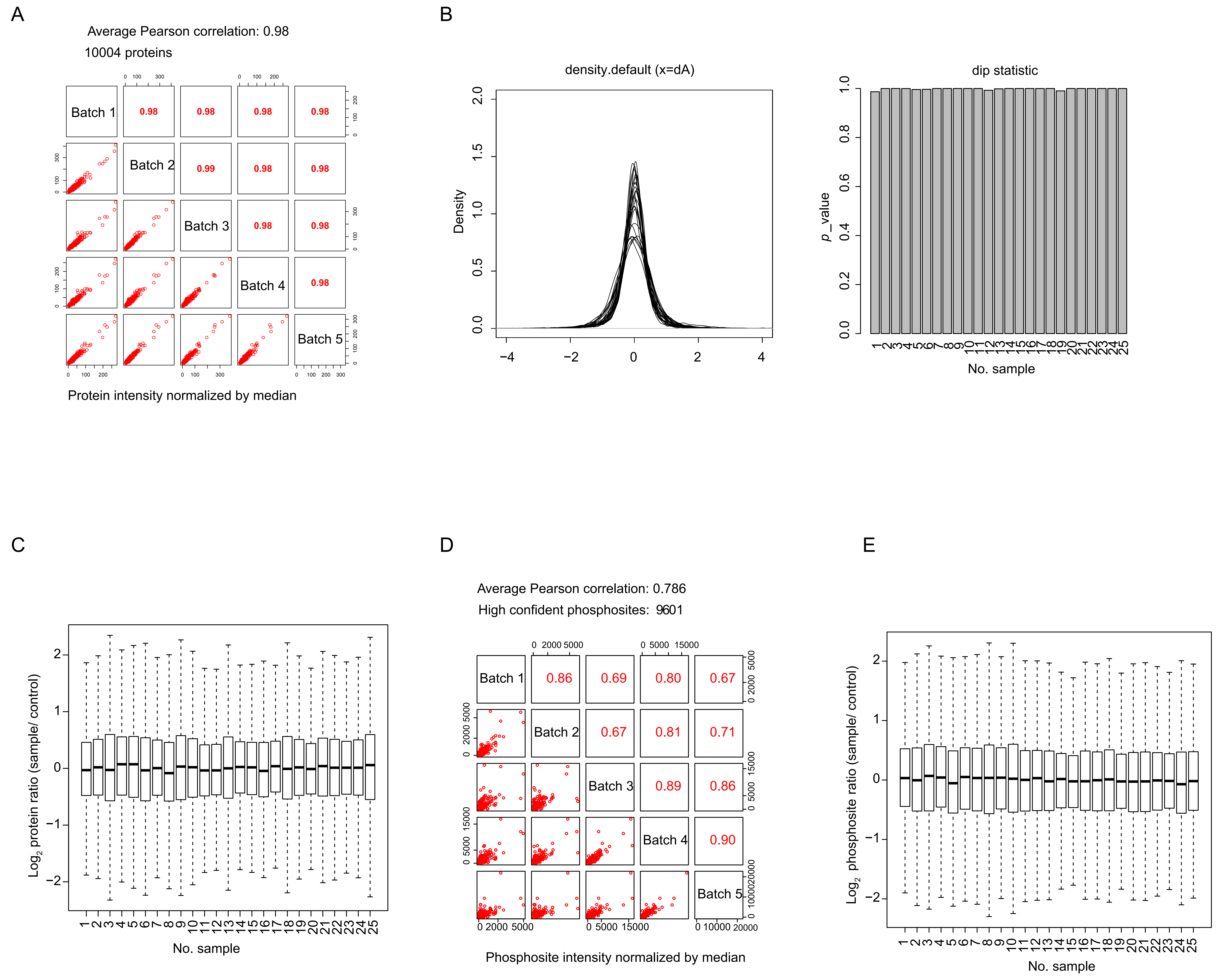

Supplementary Figure 1. Sample quality control and data normalization

A. Variation assessment between batches using protein intensity normalized by median. Scatter plots and Pearson correlations comparing five internal references from five batches were shown.

B. Quality control analysis for twenty-five tumors. The distributions of $\log_2$ protein ratio (tumor/internal reference) were assessed.

C. Log_2 protein ratios were normalized by z-score in each sample. Box plots: center lines, medians; box limits, 25th and 75th percentiles; whiskers extend to 1.5 times the IQR (interquartile range) from the box edges; dots, outliers.

D. Variation assessment between batches using phosphosites intensity normalized by median. Scatter plots and Pearson correlations comparing five internal references from five batches were shown.

E. Log_2 phosphosites ratios were normalized by z-score in each sample. Box plots: center lines, medians; box limits, 25th and 75th percentiles; whiskers extend to 1.5 times the IQR (interquartile range) from the box edges; dots, outliers.
