# Supplementary material for: Proteomic and Phosphoproteomic Maps of Lung Squamous Cell Carcinoma From Chinese Patients
Source: Front Oncol. 2020 Jun 16;10:963. doi: 10.3389/fonc.2020.00963 (PMC7308564; doi:10.3389/fonc.2020.00963)
Supplement: Supplementary file 3 [file Image_2.pdf]

Supplementary Figure 2

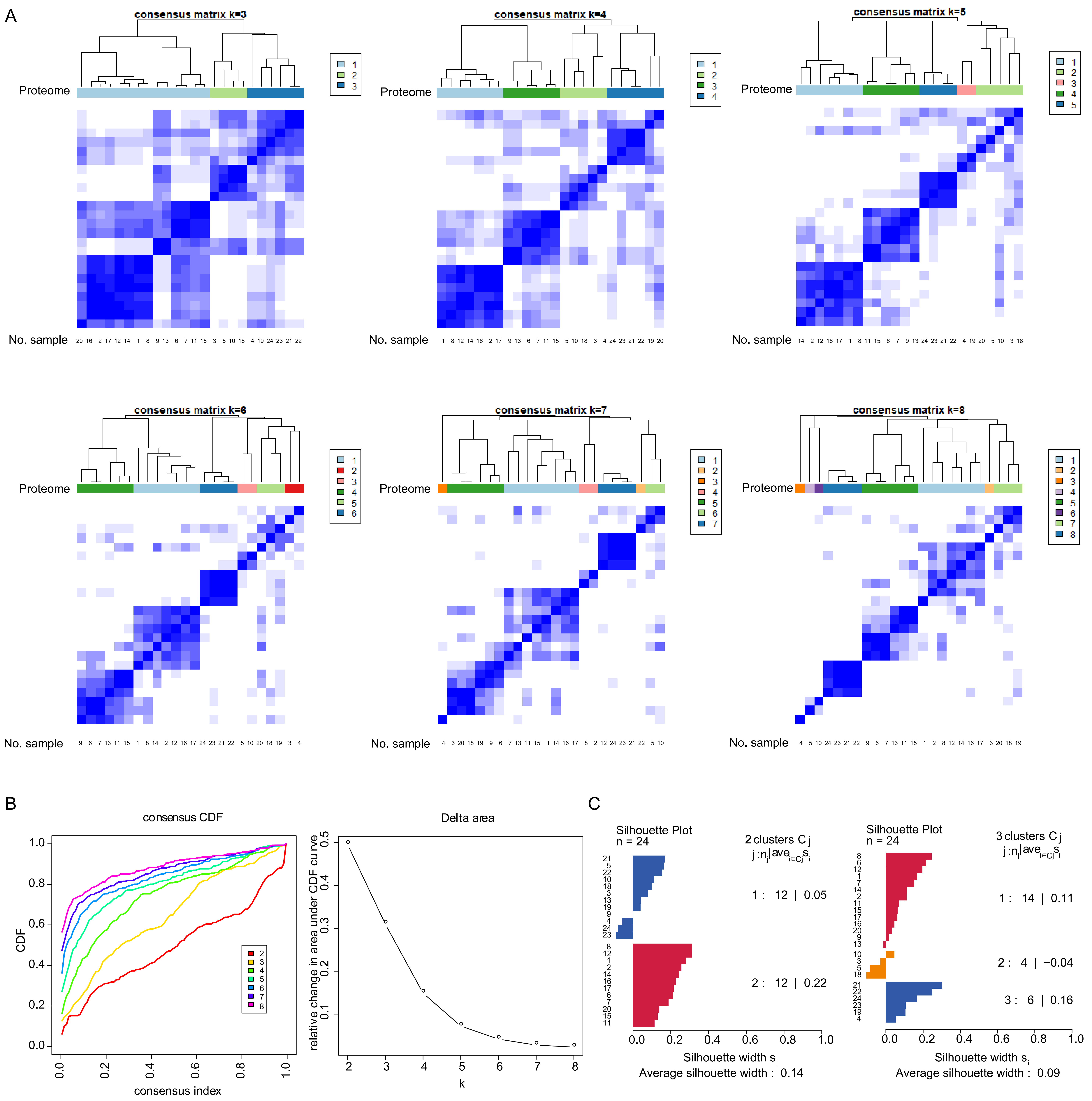

Supplementary Figure 2. Identification of optimal proteome clusters for twenty-four tumors.
A. Visualization of consensus matrices from k-means consensus clustering for *k* = 3, 4, 5, 6, 7 and 8 target clusters.
B. Consensus cumulative distribution function (CDF) area and delta area (change in CDF area) plots for 2–8 clusters.
C. Silhouette plots were generated to evaluate the coherence of the clustering for *k* = 2 and *k* = 3 clusters.
