# Supplementary material for: Proteomic and Phosphoproteomic Maps of Lung Squamous Cell Carcinoma From Chinese Patients
Source: Front Oncol. 2020 Jun 16;10:963. doi: 10.3389/fonc.2020.00963 (PMC7308564; doi:10.3389/fonc.2020.00963)
Supplement: Supplementary file 4 [file Image_3.pdf]

Supplementary Figure 3

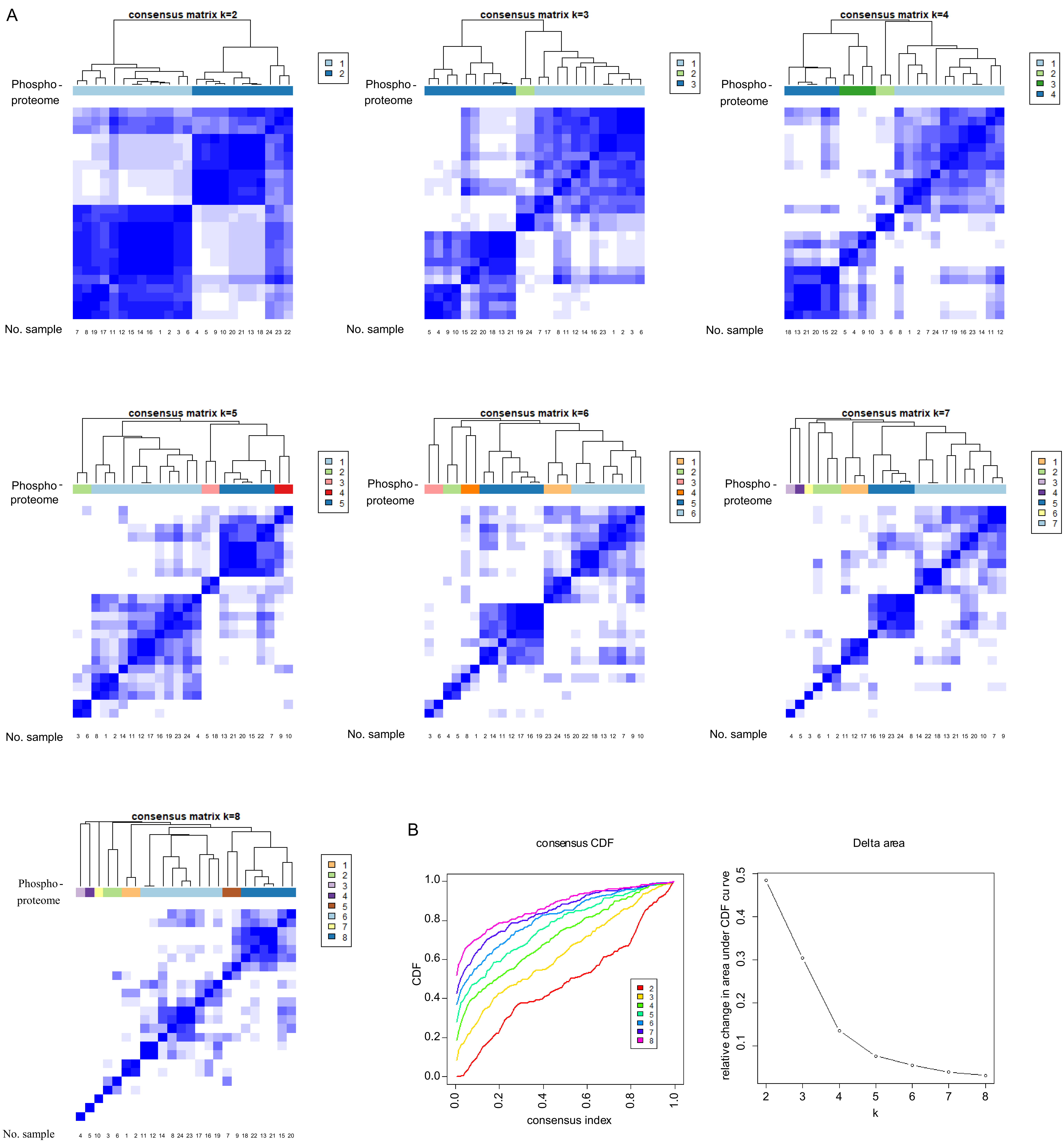

Supplementary Figure 3. Identification of optimal phosphoproteome clusters for twenty-four tumors.
A. Visualization of consensus matrices from k-means consensus clustering for $k = 2, 3, 4, 5, 6, 7$ and 8 target clusters.
B. Consensus cumulative distribution function (CDF) area and delta area (change in CDF area) plots for 2–8 clusters.
