# Supplementary material for: Proteomic and Phosphoproteomic Maps of Lung Squamous Cell Carcinoma From Chinese Patients
Source: Front Oncol. 2020 Jun 16;10:963. doi: 10.3389/fonc.2020.00963 (PMC7308564; doi:10.3389/fonc.2020.00963)
Supplement: Supplementary file 5 [file Image_4.pdf]

Supplementary Figure 4

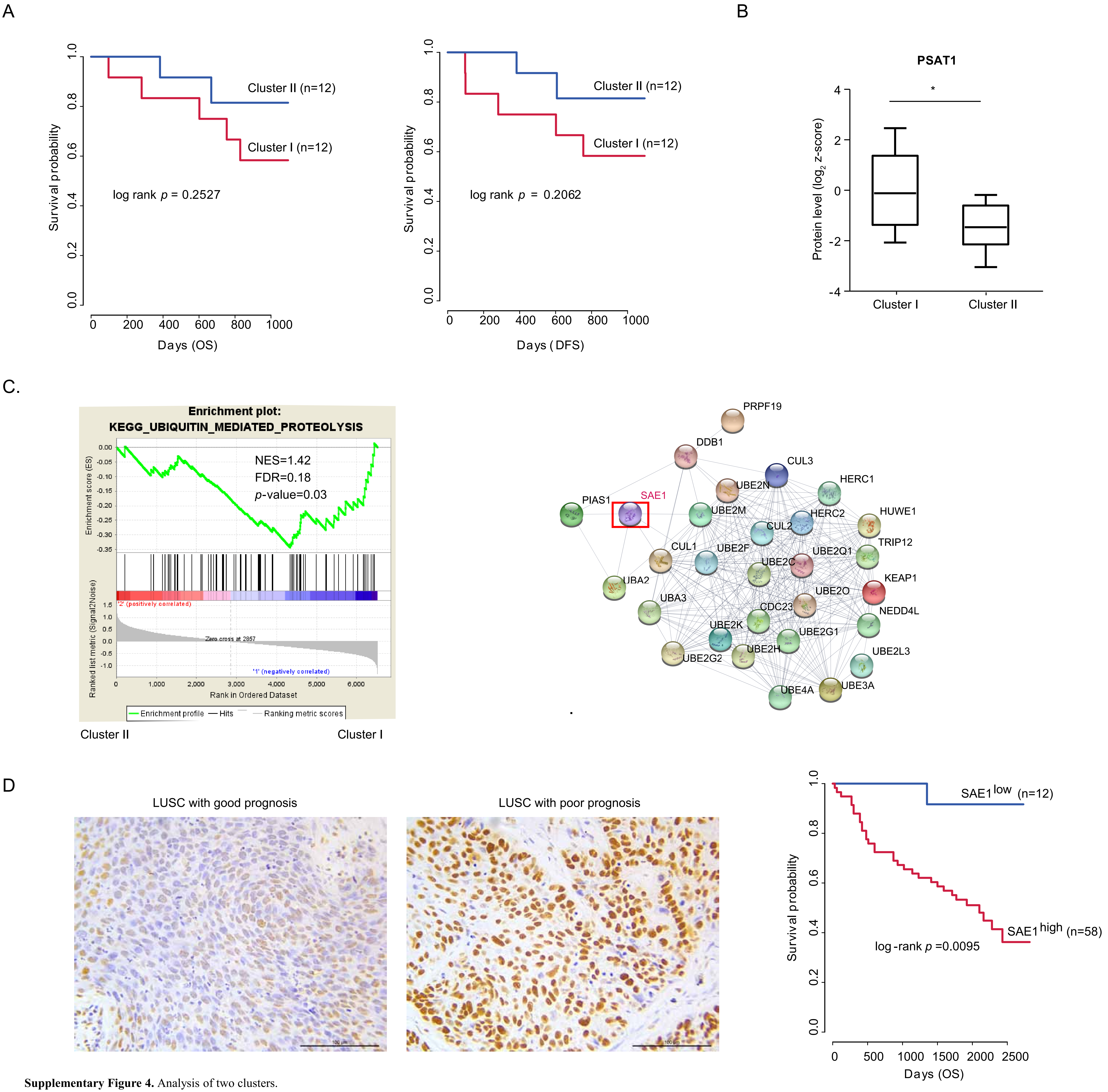

Supplementary Figure 4. Analysis of two clusters.

A. The prognosis of two clusters within 3-year follow-up.

B. PSAT1 expression between Cluster I and Cluster II.

C. Interaction of proteins that were enriched in the Ubiquitin mediated proteolysis pathway. The minimum required interaction score is 0.7 which means high confidence.

D. SAE1 expression and survival analysis in tissue microarray (TMA) assay.
