# Supplementary material for: Proteomic and Phosphoproteomic Maps of Lung Squamous Cell Carcinoma From Chinese Patients
Source: Front Oncol. 2020 Jun 16;10:963. doi: 10.3389/fonc.2020.00963 (PMC7308564; doi:10.3389/fonc.2020.00963)
Supplement: Supplementary file 6 [file Image_5.pdf]

Supplementary Figure 5

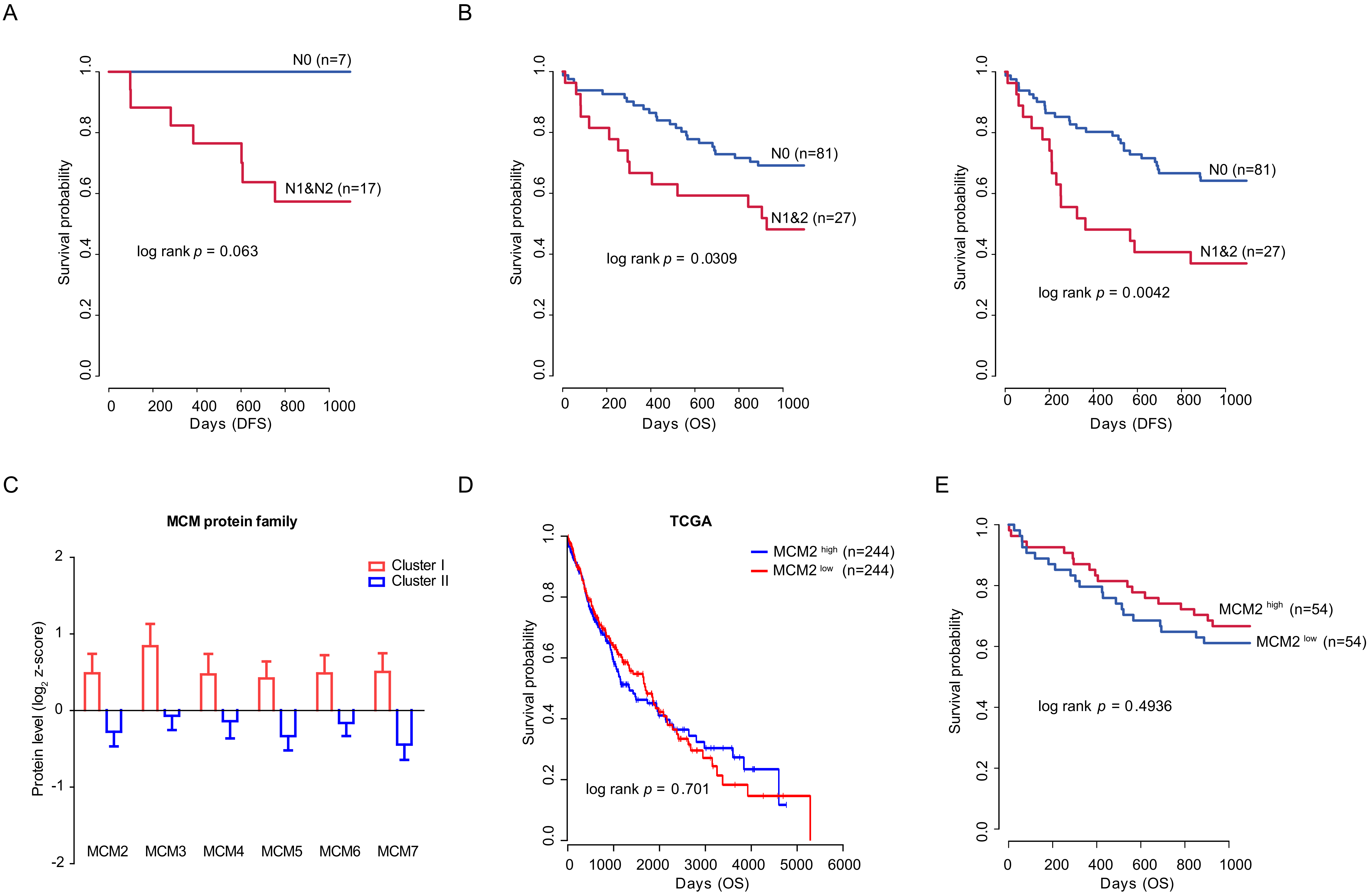

F

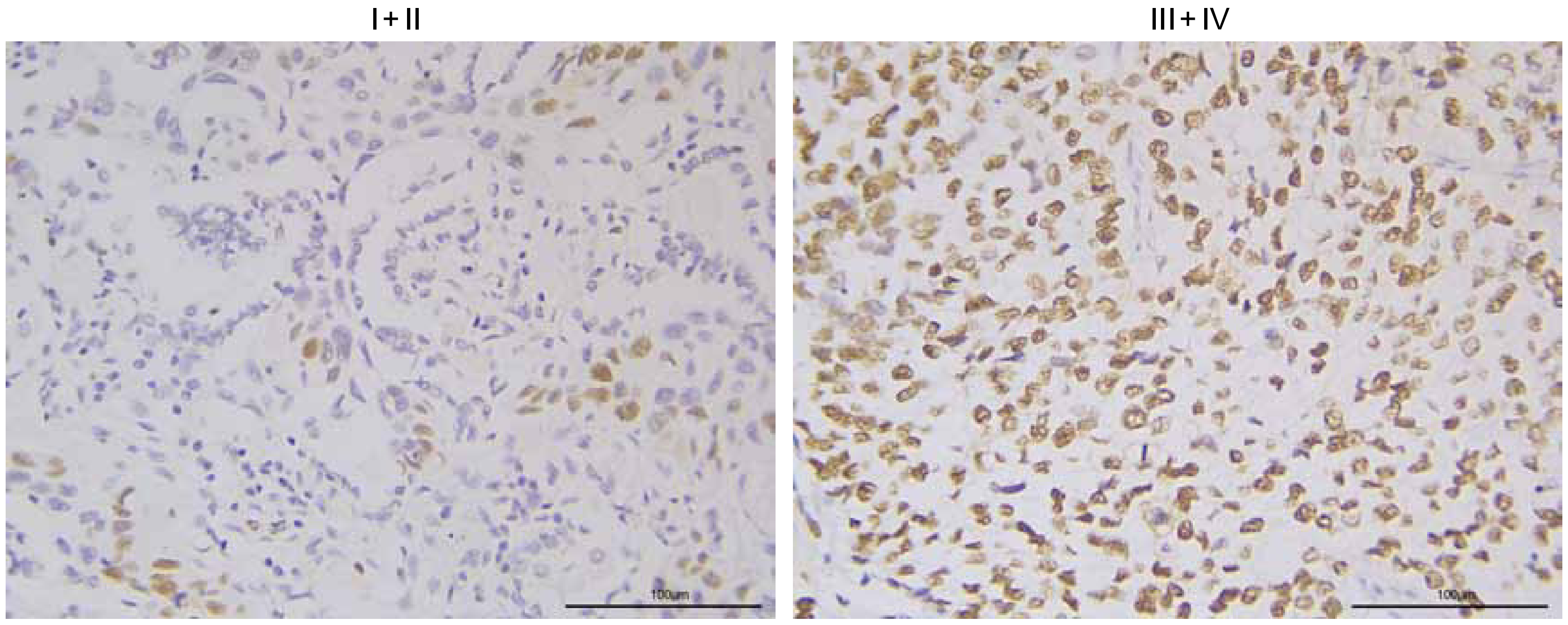

	n	MCM2 Low	MCM2 High	Pearson's χ2 text
Clinical stage				p=0. 03
I+II	39	19 (48.7%)	20 (51.3%)	
III+IV	30	7 (23.3%)	23 (76.7%)	

Supplementary Figure 5. Analysis of lymph node metastasis (LNM).
A. The prognosis of patients with or without LNM based on disease-free survival (DFS) within 3-year follow-up.
B. The prognosis of with or without LNM in previous data reported in samples from western countries within 3-year follow-up.
C. MCM protein expression in different clusters.
D. MCM2 RNA level and prognosis in LUSC (TCGA).
E. MCM2 protein level and prognosis in previous data reported in samples from western countries within 3-year follow-up.
F. MCM2 protein expression and clinical stage in tissue microarray (TMA) assay.
